# Activating Nitrogen for Electrochemical Ammonia Synthesis via an Electrified Transition-Metal Dichalcogenide Catalyst

**DOI:** 10.1021/acs.jpcc.3c08230

**Published:** 2024-04-23

**Authors:** Taylor J. Aubry, Jacob M. Clary, Elisa M. Miller, Derek Vigil-Fowler, Jao van de Lagemaat

**Affiliations:** Materials, Chemistry, and Computational Science Directorate, National Renewable Energy Laboratory, Golden, Colorado 80401, United States

## Abstract

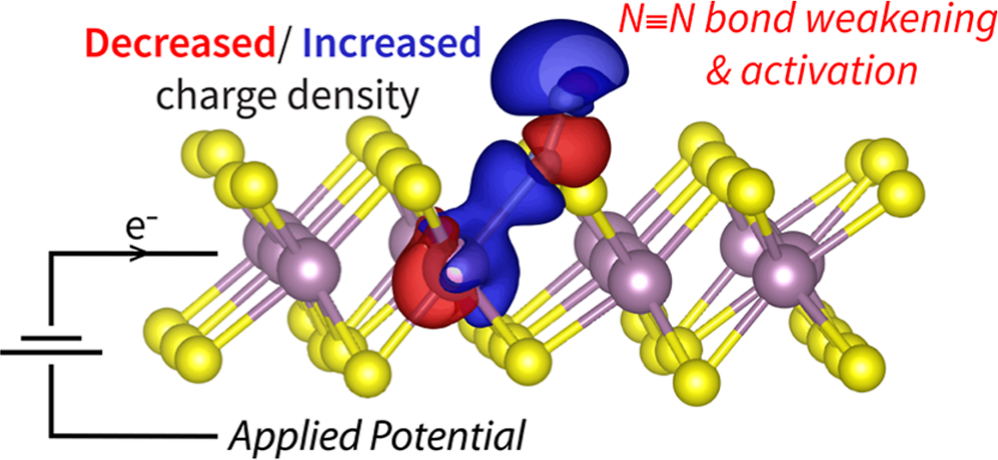

The complex interplay between local chemistry, the solvent microenvironment, and electrified interfaces frequently present in electrocatalytic reactions has motivated the development of quantum chemical methods that can accurately model these effects. Here, we predict the thermodynamics of the nitrogen reduction reaction (NRR) at sulfur vacancies in 1T′-phase MoS_2_ and highlight how the realistic treatment of potential within grand canonical density functional theory (GC-DFT) seamlessly captures the multiple competing effects of applied potential on a catalyst interface interacting with solvated molecules. In the canonical approach, the computational hydrogen electrode is widely used and predicts that adsorbed N_2_ structure properties are potential-independent. In contrast, GC-DFT calculations show that reductive potentials activate N_2_ toward electroreduction by controlling its back-bonding strength and lengthening the N–N triple bond while decreasing its bond order. Similar trends are observed for another classic back-bonding ligand in CO, suggesting that this mechanism may be broadly relevant to other electrochemistries involving back-bonded adsorbates. Furthermore, reductive potentials are required to make the subsequent N_2_ hydrogenation steps favorable but simultaneously destabilizes the N_2_ adsorbed structure resulting in a trade-off between the favorability of N_2_ adsorption and the subsequent reaction steps. We show that GC-DFT facilitates modeling all these phenomena and that together they can have important implications in predicting electrocatalyst selectivity for the NRR and potentially other reactions.

## Introduction

Electrocatalysts will play a key role in a future decarbonized economy by enabling the renewable-energy-driven conversion of small molecules such as H_2_O, CO_2_, N_2_, and CO into value-added chemicals and fuels.^1^ Developing robust catalysts that operate both efficiently and selectively has been the focus of much research and is critical for improving the sustainability of various chemical processes. The current means of ammonia production for fertilizer from N_2_ and methane, while saving billions of people from starvation, is extraordinarily energy and carbon-intensive.^2^ The Haber–Bosch process in particular currently consumes 2% of global energy production and contributes 1–2% of all greenhouse gases annually.^3^ Improving electrocatalysts to enable ammonia generation via the electrochemical nitrogen reduction reaction (NRR), in combination with green production of hydrogen, is highly desired to supplement, decentralize, and improve sustainability of fertilizer production and is of interest for carbon-free energy storage.^4,5^ However, achieving the necessary catalytic activity and selectivity toward ammonia synthesis instead of the hydrogen evolution reaction (HER) or N_2_H_4_ formation remains a major challenge for the commercial viability of electrochemical nitrogen reduction.

**Figure 1:**
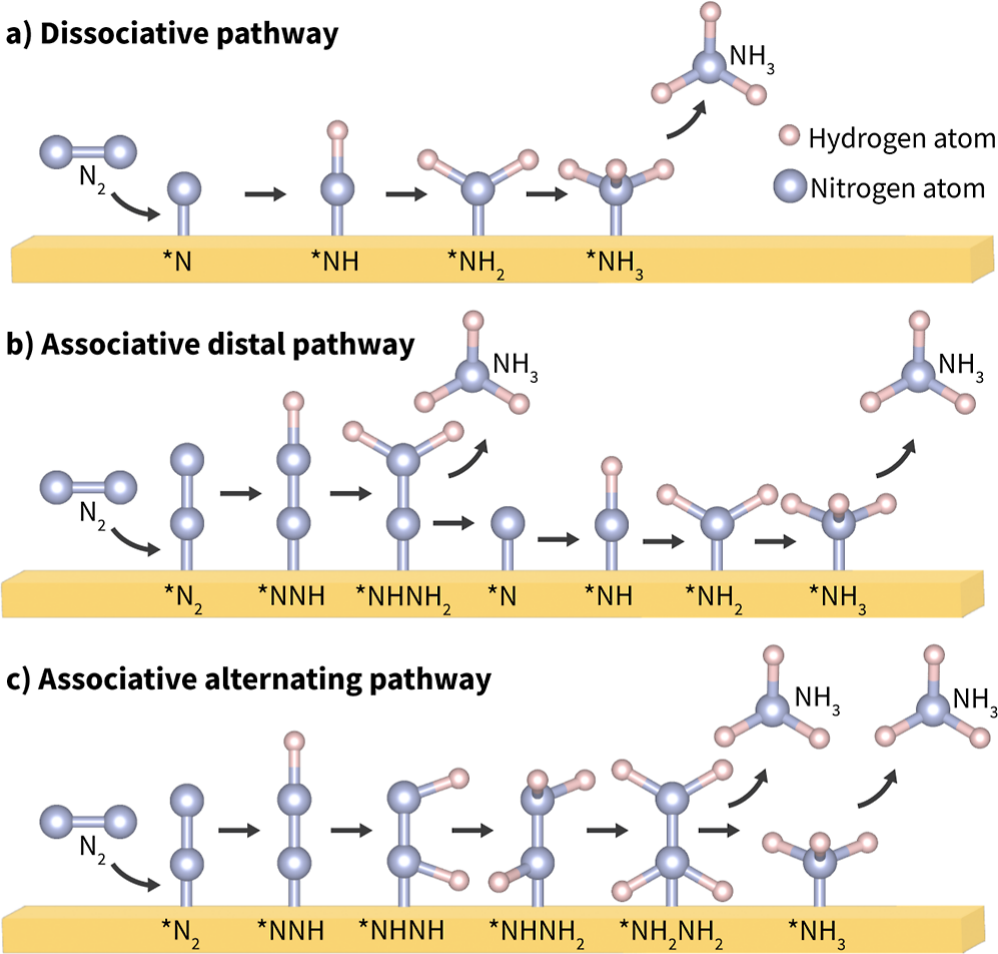
Schematic illustrations of (a) the dissociative pathway, (b) the associative distal pathway, and (c) the associative alternating pathway for the NRR on a catalyst surface, where * denotes the adsorption site on the intermediate. The solution is the proton source.

Nitrogen reduction within the Haber–Bosch process occurs via a dissociative reaction mechanism, as depicted in Figure 1a, where the strong N_2_ triple bond is broken in the first reaction step upon adsorption to the catalyst, thus requiring high temperatures and pressures around 500 °C and 200 atm.^3^ Electrochemical N_2_ reduction offers alternative routes for NH_3_ synthesis via associative reaction mechanisms in which N_2_ adsorbs to an active site and the N–N triple bond is not fully broken until the first NH_3_ is released. In both cases, the overall reaction is
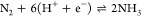
1

For a monodentate-adsorbed N_2_, the two primary associative reaction pathways involve either full hydrogenation of each N atom to NH_3_ in sequence (the “distal” pathway) or alternating hydrogenation reactions for each N atom (the “alternating” pathway), as shown in Figure 1b,c, respectively. Additionally, the NRR can proceed by a mixed pathway that shifts between the alternating and distal pathways if it is more energetically favorable to do so. Adsorption and activation of the N_2_ bond are bottlenecks in these pathways for the NRR.

Nearly all bioavailable nitrogen is formed via two processes, the industrial Haber–Bosch method and biological nitrogen fixation through nitrogenases, both of which employ transition metal-based catalysts.^6^ The NRR on nitrogenase catalysts is of particular interest because it occurs under comparatively mild biological reaction conditions. In this process, the nitrogenases’ transition metal centers adsorb N_2_ and activate it for reduction through π-backdonation.^6,7^ Here, the σ orbital of nitrogen overlaps with an empty d-orbital of a transition metal atom, and an occupied d-orbital of the transition metal atom overlaps with the empty π* orbital of N_2_. This results in the injection of electrons to the antibonding N_2_ π* orbital and weakening of the N_2_ triple bond. As such, there has been sustained research toward developing new transition metal-based catalysts that can adsorb N_2_ via a π-back-bonding mechanism, are highly active under mild reaction conditions, and have low selectivity toward the HER.^4,8^

Two-dimensional transition metal dichalcogenides (2D-TMDCs) in particular are an attractive class of materials for NRR catalysis due to their high surface area (yielding a high density of active sites), chemical tunability, and earth abundance of certain types such as MoS_2_. Many TMDCs have been studied for the NRR.^9−12^ Recently, MoS_2_ in particular has shown promise for electrochemical nitrogen reduction.^13−17^ For example, Zi et al. achieved a Faradaic efficiency of 18.9% and yield rate of 130 μg h^–1^ mg^–1^ using sulfur vacancy-rich 1T-MoS_2_.^13^ The NRR mechanism for this catalyst is believed to involve the formation of sulfur vacancies that allow favorable N_2_ adsorption near transition metal atoms. However, for this catalyst to become industrially viable, further improvements in the above metrics must still be made. Obtaining a detailed fundamental understanding of the reaction mechanism is crucial to further optimizing these electrocatalysts for the NRR and increasing their yield and efficiency.

To this end, density functional theory (DFT) has emerged as a powerful computational approach, as it can probe, among other properties, reaction energetics, catalyst selectivity, and intermediate structures at a molecular level. The modeling of electrocatalytic reactions using DFT adds challenges that are not present in gas-phase reaction models due to the complex solid–liquid interface and applied potential.^18,19^ Surface charging of the electrocatalyst and its interaction with the solvent, reaction species, and charged ions, as present in real electrocatalytic systems,^20^ can significantly affect reaction pathway energetics. As a result, simplifying trade-offs historically have been made for DFT electrocatalytic models to be computationally tractable. For example, the application of potential results in electrocatalyst charging experimentally. While a net charge can be applied to a periodic supercell if it is compensated with a uniform charged background extending throughout both the liquid and solid regions of the unit cell, this does not reflect the reality of the electrochemical system. Much more commonly, applied potential has been accounted for in DFT studies via the application of a linear postprocessing correction to the energies of uncharged canonical ensemble calculations using the computational hydrogen electrode (CHE) approach.^21^ Accordingly, this means any reaction kinetics computed in the canonical ensemble also ignores the charging effects of applied potential. In recent years, the establishment and implementation of the grand canonical DFT (GC-DFT) formalism^22−24^ has enabled the self-consistent inclusion of applied potential within each DFT calculation by allowing the number of electrons in the system to equilibrate to a set chemical potential. Thus, GC-DFT provides a more realistic and accurate approach to modeling electrocatalytic systems and has already been successfully used to study many catalysts and reactions.^25−29^ However, to the best of our knowledge, no studies have used this formalism to model this class of catalysts (TMDCs) for the NRR.

In this work, we predict electrocatalytic NRR thermodynamics on 1T′-MoS_2_ and compare the results obtained using canonical and grand canonical approaches. In particular, we focus on an active site formed by a sulfur vacancy because of the existing promising experimental research involving vacancy and defect-rich MoS_2_.^14,17^ A detailed analysis of the counteracting impacts of applied potential via stabilization of reaction intermediates and destabilization of the active site is carried out. Our results highlight the importance of using GC-DFT to accurately model electrocatalytic reactions to predict catalyst selectivity and reaction pathway energetics.

## Computational Methods

### Computational Methods to Model Applied Potential

Here, we compare the GC-DFT formalism to the traditional CHE formalism. In the CHE approach, the influence of an applied potential versus the standard hydrogen electrode (SHE), *U*, is estimated by setting the energy of an electron injected into the electrode to −*eU*. Thus, the canonical potential-dependent system free energy change in a reaction, Δ*G*(*U*), is
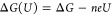
2where Δ*G* is the free energy change in a reaction using uncharged systems calculated within the canonical ensemble, *e* is the elementary charge, and *n* is the number of proton/electron pairs transferred in the reaction. Additionally, the energy of a solvated proton/electron pair at pH = 0 is taken to be in equilibrium with 1 bar H_2_ ( H_2_*⇌* H^+^ + e^–^) at 298 K, yielding
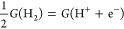
3

The CHE approach, therefore, relies on an algebraic correction to the DFT-computed energies of neutral systems. This approach has been widely applied to estimate the effect of applied potential due to its simplicity and seamless integration with traditional DFT calculations. However, there are several limitations of a fixed electron approach for electrochemistry. First, it has been shown that adsorbate interactions can vary significantly with applied potential from changes in the electronic structure, polarization from the applied field, and changes in geometry;^22,25,30^ however, the CHE approach neglects the effects of applied bias on this interface. Additionally, this formalism is limited to intermediates that are formed via proton-coupled electron transfer (PCET) steps and cannot account for noninteger changes in transferred electrons. Finally, the reaction energies along a pathway are not all calculated at a constant electron chemical potential relative to a vacuum because the Fermi energy of each charge-neutral system can vary with the element types and positions in each structure.

In GC-DFT, the grand free energy change of a reaction, Φ, is computed using
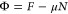
4where *F* is the Helmholtz free energy, μ is the potential relative to vacuum, and *N* is the equilibrated number of electrons and is not constrained to be an integer. The temperature, volume, and chemical potential are fixed, while the number of electrons is self-consistently varied within the part of the system that is treated quantum mechanically such that its Fermi level is in equilibrium with the applied electrochemical potential. Charge neutrality is maintained by the continuum electrolytes via adjustment of the ion concentration locally (in the electronic-DFT region) while the total ion concentration is maintained (in the classical regions).^22,23^

Additionally, the solvated proton and electron are independent references. The solvated proton can be referenced to Φ(H_3_O^+^) – Φ(H_2_O), which is not dependent on the applied potential, while the energy of an electron is referenced to *eU*_SHE_. This allows for simulation of the bias-dependence of non-PCET steps.^26,29^

### Calculation Details

All grand canonical and canonical ensemble DFT calculations were performed using the JDFTx software package (version 1.7.0)^31^ using the Perdew–Burke–Ernzerhof generalized gradient approximation functional and optimized fully relativistic norm-conserving Vanderbilt pseudopotentials^32^ from the PseudoDojo Project^33^ with stringent accuracy and 45 Ha plane-wave cutoff. The effects of spin-polarization were included; however, all calculations converged to the spin-unpolarized case. The Grimme D3 scheme was used to capture the interlayer van der Waals interactions.^34^ All calculations were performed using the minimally empirical CANDLE implicit solvation model and electrolyte concentration set to 0.5 M based on experimental conditions.^15,35^ The CANDLE model accounts for charge asymmetry by adjusting the cavity depending on the local charge environment of the solute. It has been shown to accurately reproduce solvation energies of neutral molecules, cations, and anions in water and therefore provides a computationally tractable approach to treat solvated electrochemical systems.^36^ All potentials were referenced to the calibrated absolute offset of 4.66 eV for the CANDLE model.^36^

Periodic MoS_2_ layers were separated by at least 15 Å, and coulomb truncation in the out-of-plane direction was used to prevent periodic interactions. The primitive cell of 1T-MoS_2_ was first fully optimized by using a 12 × 12 × 1 *k*-point grid. The relaxed primitive cell was expanded into a 4 × 4 × 1 supercell of 1T-MoS_2_, with a single sulfur vacancy (Sv) created to model the defect structure. We note that several computational and experimental studies have reported that the 1T-MoS_2_ phase spontaneously relaxes to the more thermodynamically stable 1T′-MoS_2_ phase.^37−39^ We observe a similar 1T to 1T′ relaxation following the introduction of a sulfur vacancy due to Mo-atom dimerization along one in-plane lattice vector (see Figure S1) and thus focus on understanding the NRR mechanism on 1T′-MoS_2_ with sulfur vacancy defects (1T′-MoS_2_-Sv). For the supercell, a 3 × 3 × 1 *k*-point grid was used, and all atoms were again allowed to relax. The force and energy optimizations were converged to within 10^–4^ Ha/Bohr and 10^–5^ Ha, respectively. Initial adsorption structures were generated using Pymatgen.^40^ After full relaxation of the vacancy-defected MoS_2_ structure, to eliminate unphysical periodic distortions in the MoS_2_ layer, only adsorbate and MoS_2_ atoms within a 3.5 Å radius from the sulfur vacancy center were allowed to relax (see Section S1.2 on selection of the relaxation radius). Where noted, vibrational corrections to the free energy were made using the vibrational free energy calculated using the vibration module in JDFTx at 298 K. The free energy correction for the concentration dependence of a proton [*G*(H^+^) = −*kT* × ln 10 × pH]^21,41^ is small at the low pHs used for NRR (≤−0.06 eV for pH ≤ 1) and is therefore omitted. Converged structures and standardized calculation input files from this work can be found at https://github.com/tayloraubry/MoS2-NRR-data.

Net atomic charges and bond orders were computed using the density-derived electrostatic and chemical (DDEC6) method using the Chargemol program (version 09-26-2017),^42−44^ which involves spherical averaging of the atomic electron density obtained from the DFT calculations. Previous work has shown that the DDEC6-derived bond orders exhibit good reliability across a broad range of materials.^45^ See the Supporting Information for additional details related to the orbital projected density of states (pDOS) and crystal orbital Hamilton population (COHP) analysis.

To calculate the *NN to *NNH transition states at the extremes of potential in this study, we utilized a previously developed grand canonical implementation of the nudged elastic band method (GC-NEB)^26^ that utilizes JDFTx within the pythonic atomic simulation environment (ASE)^46^ to enableuse of the ASE implementation of the NEB method.

## Results and Discussion

### Catalytically Active Sites

A first step toward understanding how the NRR proceeds on 1T′-MoS_2_ is to identify the active sites. While not the only descriptor of activity, in order for a catalyst to be active, it must bind the reactant. The first step of the NRR involves N_2_ binding and activation, which is notoriously difficult due to N_2_ having a full valence shell, strong triple bond, and no bond polarization,^6,47^ and therefore is often a bottleneck for the NRR.^48^ Not surprisingly, unlike for the HER,^49,50^ the fully coordinated surface sites of MoS_2_ are inactive for NRR and exposed Mo atom sites are needed to bind N_2_. We find that both vacancies and edges on 1T′-MoS_2_ are capable of binding to N_2_. Here, we focus on vacancy defects in view of promising results from the literature for NRR from vacancy and defect-rich MoS_2_.^14,17^

**Figure 2:**
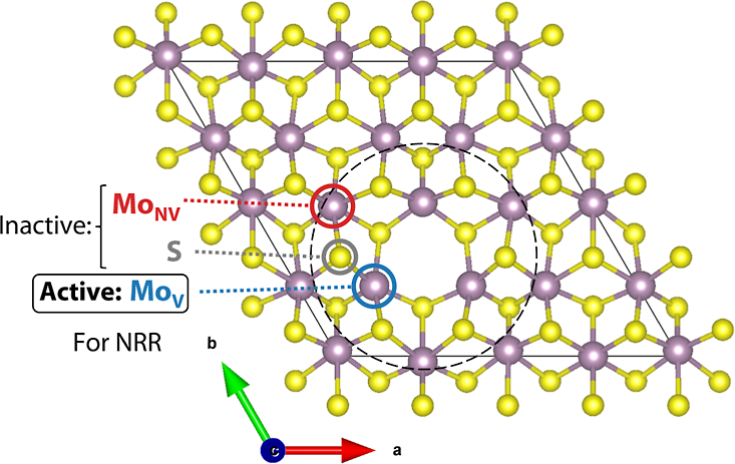
Top-down view of the structure used to model 1T′-phase MoS_2_ with sulfur vacancy defects (1T′-MoS_2_-Sv) in which sulfur atoms are represented by yellow spheres and molybdenum atoms are represented by purple spheres. Surface sulfur (S, gray label) and fully coordinated nonvacancy molybdenum (Mo_NV_, red label) sites do not bind nitrogen and are therefore inactive for the NRR. Exposed molybdenum sites at edges or vacancies are needed to bind N_2_. This work computes the NRR reaction pathway at a molybdenum vacancy (Mo_V_, blue label). The vacancy structure initially was allowed to fully relax and the black dashed line shows the 3.5 Å radius of atoms that were allowed to relax for subsequent adsorption calculations to prevent unphysical distortions in the lattice (see Calculation Details).

MoS_2_ is naturally sulfur vacancy defect rich, with chemical vapor deposition and exfoliation methods yielding defect densities of the order 1 × 10^13^ cm^–2^.^51,52^ Additionally, there are several methods to experimentally tune the sulfur defect density of MoS_2_, including modifying the growth conditions,^53^ postgrowth desulfurization techniques such as Ar plasma exposure,^54^ H_2_ annealing,^55^ and electrochemical desulfurization (at reductive potentials of at least −1 V vs RHE),^56^ as well as postgrowth defect healing.^57^Figure 2 shows the model system for this work and denotes the molybdenum nonvacancy (Mo_NV_) and surface sulfur sites (S) that we determined are inactive. We sampled several N_2_ configurations within the sulfur defect (in solution without applied potential, see Figure S3 and Table S2 for surface sampling data) and found that N_2_ preferentially binds in an end-on configuration with equivalent binding energies for symmetrically equivalent molybdenum vacancy (Mo_V_) sites. Under implicit solvation with no applied potential, the adsorption energy of N_2_ to the Mo_V_ site is −0.31 eV, and we observe an elongation of the N_2_ triple bond to 1.118 Å from our calculated value of 1.096 Å for free molecular nitrogen, which closely matches the literature value of 1.098 Å.^6,47^

### Effect of Potential on N_2_ Adsorption and Activation

**Figure 3:**
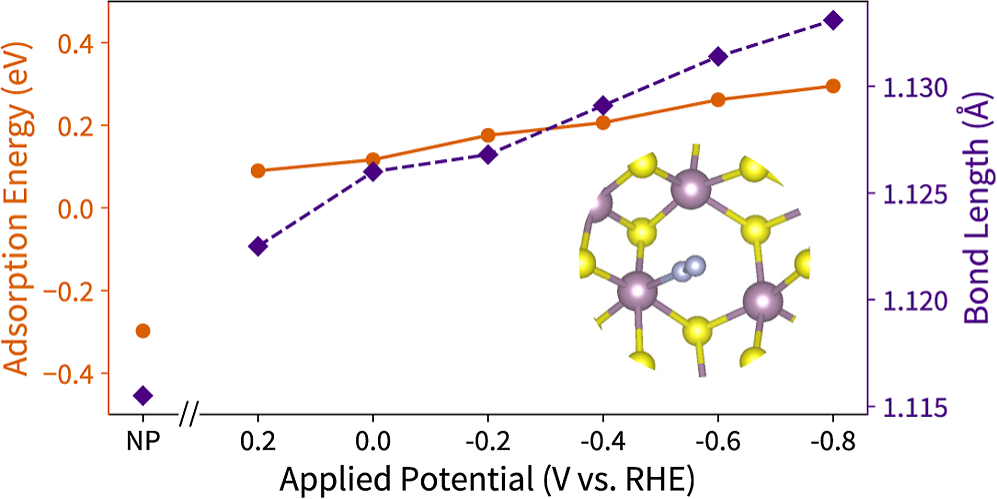
N_2_ adsorption energy (orange circles) and N–N bond length (purple diamonds) under solvation with no potential in the canonical ensemble (NP) and applied potentials using GC-DFT. The adsorption energy goes from favorable (negative) without an applied potential to unfavorable (positive) under all applied potentials (−0.8 to +0.2 V). The N–N bond length simultaneously increases as more negative potentials are applied, indicating activation of N_2_.

To understand the effects of applied potential and surface charging on the NRR as would be present in an electrochemical system, we performed GC-DFT calculations at experimentally relevant potentials (−0.8 to +0.2 V vs RHE).^13,35,58^ Our calculated N_2_ adsorption energies and bond lengths are shown in Figure 3. Since N_2_ binding does not involve a PCET, the CHE approach predicts that its adsorption energy is independent of potential. In contrast, our GC-DFT calculations predict that the adsorption energy of N_2_ is potential dependent. Surprisingly, N_2_ binding becomes less favorable for more reductive potentials, which is likely to impede the NRR even though the driving force for electroreduction is increased, while the N–N bond concomitantly lengthens, which indicates bond weakening and N_2_ activation. We can begin to understand these seemingly conflicting results by using potential-dependent system bond orders, charge densities, and electronic structures to further characterize the role of Mo back-bonding in N_2_ binding.

**Figure 4:**
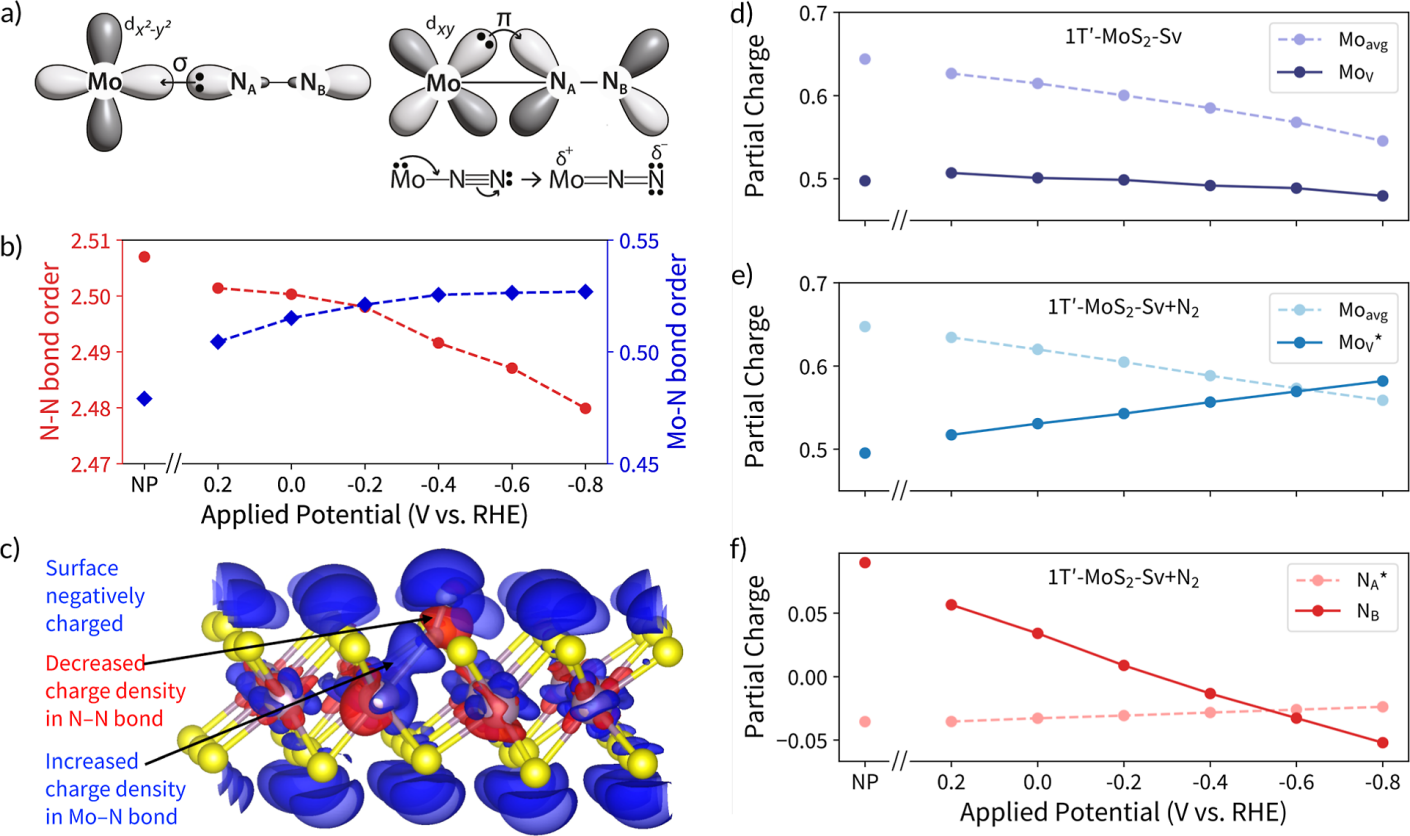
(a) Orbital picture of the π-back-bonding mechanism. (b) Bond order under solvation with no potential in the canonical ensemble (NP) and as a function of potential using GC-DFT for the N–N bond (red circles) and the Mo–N bond (blue diamonds) in the 1T′-MoS_2_-Sv + N_2_ structure calculated using the DDEC6 method. By the same method, the isolated N_2_ molecule bond order is 2.8. (c) Charge density difference of the 1T′-MoS_2_-Sv + N_2_ structure at the ±0.0001 e/Å^3^ isosurface level for a −0.2 V step toward negative potential (the 0.0 to −0.2 V step is shown; however, similar results were observed across all potential steps). Red indicates regions of charge loss and blue indicates regions of charge gain. (d–f) Partial charges on selected atoms in (d) 1T′-MoS_2_-Sv and (e,f) 1T′-MoS_2_-Sv with N_2_ adsorbed. Mo_avg_ denotes the average for all molybdenum atoms, Mo_V_ denotes the specific molybdenum vacancy active site denoted in Figure 2, and the nitrogen atoms in the adsorbed structure are denoted by N_A_ and N_B_. In all cases, * denotes an adsorption site.

In the typical π-back-bonding mechanism, depicted in Figure 4a, the bonding nitrogen (*N_A_) donates electrons to the metal center through a σ bond, while the occupied d-orbital of Mo injects electrons into the antibonding π* orbital of N_2_. This should lead to a nonzero Mo–N bond order and a decrease of the N–N bond order from its predicted desorbed molecular value of 2.8. Indeed, we predict that each of these occurs both with and without the presence of an applied potential (Figure 4b). However, the specific impact of additional electrons present due to the use of more reducing potentials is dependent on system chemistry. To visualize this, the impacts of decreasing the applied potential by 0.2 V versus RHE on the bound 1T′-MoS_2_-Sv + N_2_ structure charge density are shown in Figure 4c. It shows that for a decreased applied potential: (1) the MoS_2_ basal plane and outer N atom (N_B_) become more negatively charged (blue lobes on the top and bottom of the MoS_2_ layer), (2) there is increased charge density in the Mo–N bond, and (3) there is a decrease in charge density in the N–N bond. These observations were consistent across the entire potential range used here. Additionally, the calculated 1T′-MoS_2_-Sv + N_2_ charge densities for each applied potential can be partitioned into atomic charges by using the DDEC6 method. This partitioning shows that, on average, the Mo atoms in the 1T′-MoS_2_-Sv and 1T′-MoS_2_-Sv + N_2_ structures lower their charge state with a more negative potential (Figure 4d,e). However, the charge state for the Mo_V_ atom with adsorbed N_2_ increases while the charge state for the Mo_V_ atom in the neat structure decreases (Figure 4d,e). This is accompanied by a decrease in the outer nitrogen (N_B_) charge state while the bonded nitrogen remains at a relatively more constant charge (Figure 4f).

The negative charging of the MoS_2_ basal plane and average Mo atom is expected because the point of zero charge (PZC), or the potential at which the system is uncharged, for the 1T′-MoS_2_-Sv + N_2_ structure is +0.61 V vs RHE. Similarly, the decrease (increase) in N–N (Mo–N) charge density with more negative potentials is consistent with the decreasing N–N (increasing Mo–N) bond order shown in Figure 4b and the increasing (decreasing) bond lengths of each bond shown in Figures 3 and S4. These opposite trends in Mo–N and N–N bond order indicate that the destabilization of N_2_ with reductive potentials likely arises from a combination of factors, and is not solely related to the change in charge density for a single bond, helping to explain why the N_2_ remains adsorbed despite the unfavorable adsorption energies. All of the above discussion is also consistent with the changes in the atom charge state of the Mo and N atoms involved in N_2_ binding. Taken together, we conclude from these results that N_2_ binds to this active site via π-back-bonding with Mo and that this mechanism is affected by the use of a self-consistent applied potential within GC-DFT. Next, we show how the inclusion of additional NRR intermediates in the reaction thermodynamics is important in order to explain why reductive potentials are so necessary to drive this reaction in the literature.

### Comparison of NRR Path under Applied Potential in the CHE and GC-DFT Approaches

**Figure 5:**
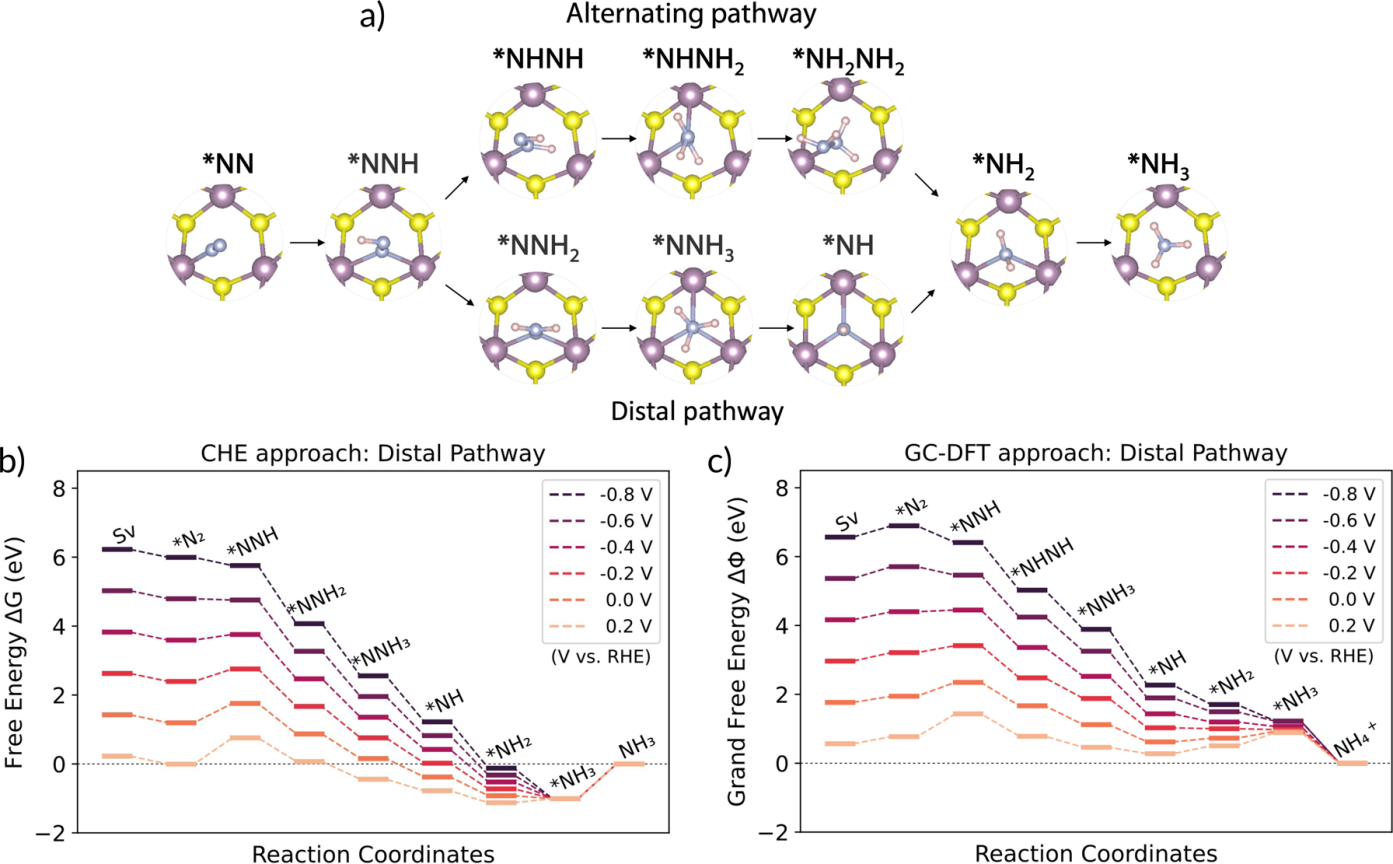
Reaction pathways for NRR at Mo_V_ site of 1T′-MoS_2_-Sv. (a) The calculated intermediate structures for both the alternating (above) and distal (below) pathways. The energetics for the distal reaction pathway in the (b) CHE and (c) GC-DFT approach. The energetics for the alternating pathway are shown in Figure S6.

Our calculations for the full NRR reaction pathway at the Mo_V_ site of 1T′-MoS_2_-Sv are summarized in Figure 5. The structures of the optimized intermediates for both the alternating and the distal pathways are shown in Figure 5a. For simplicity, we focus on the distal pathway which has the lower overall potential determining steps (PDSs) compared to the alternating pathway shown in the Supporting Information. Figure 5b,c shows the energetics of the NRR in the CHE and GC-DFT approaches with vibrational corrections included. At the desorption steps, NH_3_ can be desorbed or further protonated and desorbed as NH_4_^+^, especially in acidic media. In the final desorption step, an endothermic Δ*G* is commonly obtained for NH_3_ desorption, and it has been noted that further protonation to NH_4_^+^ would cause the adsorbate to be easily released into the solution.^59−61^ In the GC-DFT approach, NH_4_^+^ desorption in the final step can be explicitly calculated by referencing molecular NH_4_^+^ as shown in Figure 5c yielding favorable NH_4_^+^ desorption. We note that it is possible to reference NH_4_^+^ in the CHE method using its standard reduction potential;^29,62^ however, this method is not yet commonly used in the literature. We instead reference NH_3_ for better comparison but do not consider this a PDS. The full chemical reactions for each step of the pathways used in our calculations are shown in eqs S1.1–S2.8. Results for the alternating pathway are shown in Figure S6. Additionally, GC-DFT pathways referenced to NH_3_ and all pathways without vibrational corrections are shown in Figures S6 and S7, respectively.

In comparing the pathways for the two computational approaches, it is readily noticeable that they predict different PDSs. In the CHE approach, *N_2_ adsorption is always favorable, while the *NNH step is the PDS until reductive enough potentials are applied. Similarly, in the GC-DFT approach, the *NNH step is the PDS until reductive enough potentials are applied; however, these potentials simultaneously destabilize N_2_ adsorption. The *N_2_ to *NNH step switches from being endergonic to exergonic around −0.5 V vs RHE. The slow kinetics of the first protonation step is often identified as a barrier to efficient electrochemical NRR.^26,28^ To understand the effects of the applied potential on the kinetics, we performed GC-NEB calculations to calculate transition state pathways at the extremes of our potential ranges. Structures of the NEB images and pathway energetics are shown in Figures S9 and S10, respectively. These calculations show that the activation energy of this step is reduced from +0.6 eV at +0.2 V down to only +0.1 eV at −0.8 V which trends with more favorable thermodynamics. This suggests that the reaction could proceed at moderate temperatures and/or pressures and that more reductive potentials make the reaction more kinetically favorable. The more thermodynamically favorable protonation under more reductive potentials can be understood by the above discussion, which shows that the outer nitrogen takes on a more negative partial charge, thus becoming polarized and making the subsequent protonation easier. We also note that the selected potential range charges the system negatively for all pathway states (Figure S11 shows the calculated number of electrons at each step), indicating that the PZC is always more positive than 0.2 V vs RHE. These results highlight the importance of modeling the potential using the more physically realistic GC-DFT method, especially when complex chemical bonding is present that can be affected by the applied potential in contrasting ways.

### Diatomic Bonding to the Active Site and HER Competition

**Figure 6:**
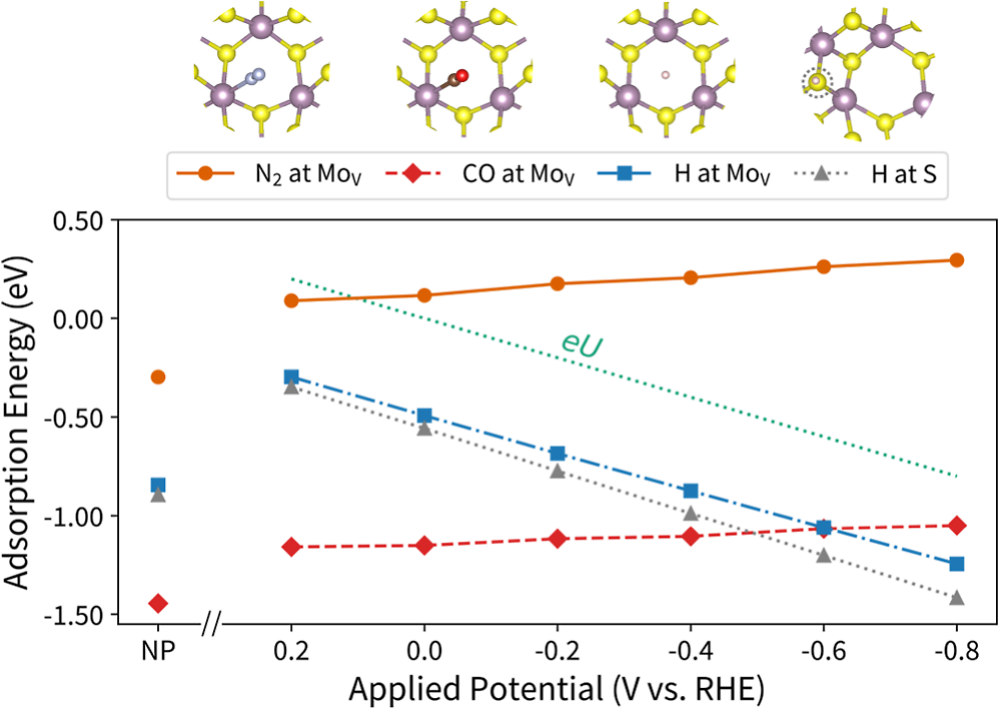
Adsorption energy as a function of potential conditions for N_2_ (orange circles), CO (red diamonds), and H (blue squares) initialized at the Mo_V_ site as well as H initialized at a surface S site (gray triangles). The structural images depict the relaxed structures, which are similar across potentials. Both the N_2_ and CO GC-DFT adsorption energy data follow the same trend and are destabilized for more negative potentials while the H GC-DFT adsorption energies are stabilized for more negative potentials. A (dotted green) line represents the elementary charge multiplied by the applied potential (*eU*), the CHE potential correction, which is overlaid to show that its slope is similar to that of the GC-DFT H adsorption data.

To extend our results beyond nitrogen, we also calculated the potential-dependent GC-DFT adsorption energies of CO, which is an important intermediate in CO_2_ reduction and the textbook example of a backbonding molecule, and H, which is not only important for the HER but also a deleterious competing reaction for the NRR. These results are plotted alongside N_2_ in Figure 6. The slope of the GC-DFT H adsorption energies for different potentials is close to the slope of the CHE approach’s *eU* correction, indicating that, in this case, the CHE approach is a good approximation for describing the potential dependence of H adsorption energies. This occurred because the neat 1T′-MoS_2_-Sv system’s and 1T′-MoS_2_-Sv + H systems’ GC-DFT charge states happened to differ by 1 electron on average for each potential in the range studied here (0.94 for H at Mo_V_ site and 1.05 for H at S site), meaning this adsorption reaction is reasonably described by a single PCET within the CHE approach. However, because the 0 V vs RHE H adsorption energies differ significantly between the CHE and GC-DFT approaches (−0.84 vs −0.49 eV at the Mo_V_, respectively, since – *neU* = 0 eV for U = 0 V vs SHE), we stress that the surface charging seamlessly handled by using GC-DFT can still significantly alter the predicted adsorption energies of even an adsorbate with simple bonding such as H. Like for N_2_, the CHE approach also predicts that the CO adsorption energies do not depend on potential since a PCET step is not involved in its adsorption. Although the CO molecule is more strongly bound than N_2_, we observed similar trends in the CO adsorption energy destabilization with more negative applied potentials. The relaxed CO adsorbed structures and charge density difference image for 1T′-MoS_2_-Sv + CO from a −0.2 V potential step are shown in Figure S12. A nearly identical picture is observed for CO as for N_2_, with a decreased charge density in the C–O bond and increased charge density in the Mo–C bond, indicating a similar backbonding picture. We conclude that the CHE correction can sometimes predict a similar adsorption energy potential dependence as GC-DFT calculations, such as for H adsorption over a particular potential range but can also significantly deviate from GC-DFT predictions, particularly for systems involving the more complicated orbital interactions of back-bonded molecules.

In order to gain a deeper understanding of the orbital interactions involved in bonding, we analyzed the pDOS of all of the adsorption structures shown in Figure 6 and performed COHP analysis of the 1T′-MoS_2_-Sv + N_2_ (see Figures S13–S15 and surrounding text). This analysis confirms that the GC-DFT applied potential impacts the Mo 4d and N 3σ hybridization; we observe changes in the overlap of these states for 1T′-MoS_2_-Sv + N_2_ that correlate with binding favorability. We conjecture that the large spread in energy of Mo 4d states is key to their ability to interact with bonding and antibonding states of backbonding molecules well above and below the Fermi energy.

More broadly, our finding that the GC-DFT adsorption energies can trend with opposite gradients as a function of potential has important implications for the prediction of this catalyst’s selectivity toward the NRR over the HER. Often, the selectivity of a particular catalyst is determined by comparing the adsorption energies of two species without considering the applied potential (even within the CHE approach).^54,63^ Our results show that selectivity can change at potentials where adsorption is equally favorable and crossover occurs (consider the adsorption energies of CO and H vs potential in Figure 6). Indeed, Choi et al. have observed exactly this crossover effect between H and N_2_ on a FeN_4_-based M–N–C single-atom catalyst.^60^ By linear extrapolation, we find that the crossover potential for N_2_ and H occurs at +0.5 V vs RHE using GC-DFT. Unfortunately, the much stronger binding of H to N_2_ on this active site indicates that the well-known competitive HER issue for electrochemical ammonia production is predicted to be worse for this site using the GC-DFT approach. Nevertheless, we believe that the GC-DFT approach will enable further optimization of this catalyst because it fully integrates fundamental electrochemical phenomena within its formalism. Future studies could further understand this problem using GC-DFT to probe alternative active sites and the role of explicit water molecules in N_2_ and H adsorption. Additionally, it has been suggested that catalyst selectivity toward ammonia can be improved by reducing the concentration of protons in the bulk solution to allow N_2_ to more easily compete with H for surface active sites,^64^ thus, future work incorporating larger pH ranges will also be insightful.

## Conclusions

Overall, we present an in-depth study of the NRR at sulfur vacancy defects in MoS_2_ under an applied potential. We have found that the application of reductive potentials activates N_2_ toward electroreduction by weakening the N–N triple bond, as indicated by bond lengthening and a reduction in bond order. These factors, alongside the trends in net atomic charges, strongly indicate the occurrence of back-bonding between Mo d-states and N_2_. This mechanism leads to the outer nitrogen acquiring a more negative partial charge with increasingly reductive potentials, thereby favoring the subsequent *NNH protonation step, both thermodynamically and kinetically, along with subsequent reaction steps becoming thermodynamically favorable. However, despite the increase in the Mo–N bond order, the N_2_ structure is also destabilized at more negative potentials, indicating that other factors within the structure contribute to this destabilization. This trade-off results in a balance between N_2_ adsorption and the latter reaction steps being favorable. In contrast, the CHE method predicts that only the *NNH step is the PDS, as in the model N_2_ adsorption is potential-independent. These results highlight that the effects of electrode charging due to applied potential can impact reaction energetics, leading to different PDSs. A design principle could be to target catalysts with a lower PZC so that N_2_ remains favorably adsorbed under the potentials necessary to drive electroreduction.

Similar trends in adsorption energy across potentials were observed for CO; binding becomes less favorable with more reductive potentials, with a concomitant reduction in charge density within the C–O bond. This trend is opposite to that observed with hydrogen: its adsorption energy is stabilized for more reductive potentials. The fact that these adsorbates can trend in opposite directions as a function of potential has implications for how catalyst selectivity is determined theoretically in that the selectivity determined under a vacuum or without applied potential may not hold true across different potentials. A key takeaway from these results is that a more realistic treatment of potential using GC-DFT can predict significantly different chemical behavior compared to canonical ensemble calculations, particularly when relatively complex chemical bonding is present.

## Supporting Information Available

Structural relaxation and adsorption site sampling, chemical equations for NRR pathways, calculated NRR pathways, GC-NEB calculations for the *N_2_ to *NNH step, calculated number of electrons in NRR reaction pathways, structures and charge density difference plot for CO adsorption, density of states and COHP analysis, and additional references (PDF)
