# Supplementary material for: Activating Nitrogen for Electrochemical Ammonia Synthesis via an Electrified Transition-Metal Dichalcogenide Catalyst
Source: J Phys Chem C Nanomater Interfaces. 2024 Apr 23;128(17):7063–72. doi: 10.1021/acs.jpcc.3c08230 (PMC11075086; doi:10.1021/acs.jpcc.3c08230)
Supplement: Supplementary file 1 — jp3c08230_si_001.pdf [file jp3c08230_si_001.pdf]

Supplementary information

Activating Nitrogen for Electrochemical Ammonia Synthesis via an Electrified Transition Metal Dichalcogenide Catalyst

Taylor J. Aubry,* Jacob M. Clary, Elisa M. Miller, Derek Vigil-Fowler,* and Jao van de Lagemaat*

Materials, Chemistry, and Computational Science Directorate, National Renewable Energy Laboratory, Golden 80401, CO, United States of America

E-mail: Taylor.Aubry@nrel.gov; Derek.Vigil-Fowler@nrel.gov; Jao.vandeLagemaat.gov

Contents

S1 Structural relaxation and adsorption site sampling . . . S2

S1.1 Vacancy Relaxation . . . S2

S1.2 Selection of Relaxation Radius . . . S3

S1.3 Adsorption site sampling . . . S4

S2 Chemical equations for NRR pathways . . . S5

S2.1 Standard pathways with NH_3 desorption . . . S5

S2.2 Pathways modified for ${NH_4}^+$ desorption in the final step . . . S6

S3 Calculated NRR pathways . . . S7

S4 GC-NEB calculations for the *N_2 to *NNH step . . . S10

S5 Calculated number of electrons in NRR reaction pathways . . . S11

S6 Structures and charge density difference plot for CO adsorption . . . S11

S7 Density of States and Crystal Orbital Hamilton Population analysis . . . S12

References . . . S16

S1 Structural relaxation and adsorption site sampling

S1.1 Vacancy Relaxation

The 4x4 supercell of 1T-MoS_2 was fully relaxed upon the introduction of a sulfur vacancy, which caused Mo-atom dimerization along one in-plane lattice vector as expected for a 1T to 1T′-phase change and is illustrated in Figure S1.

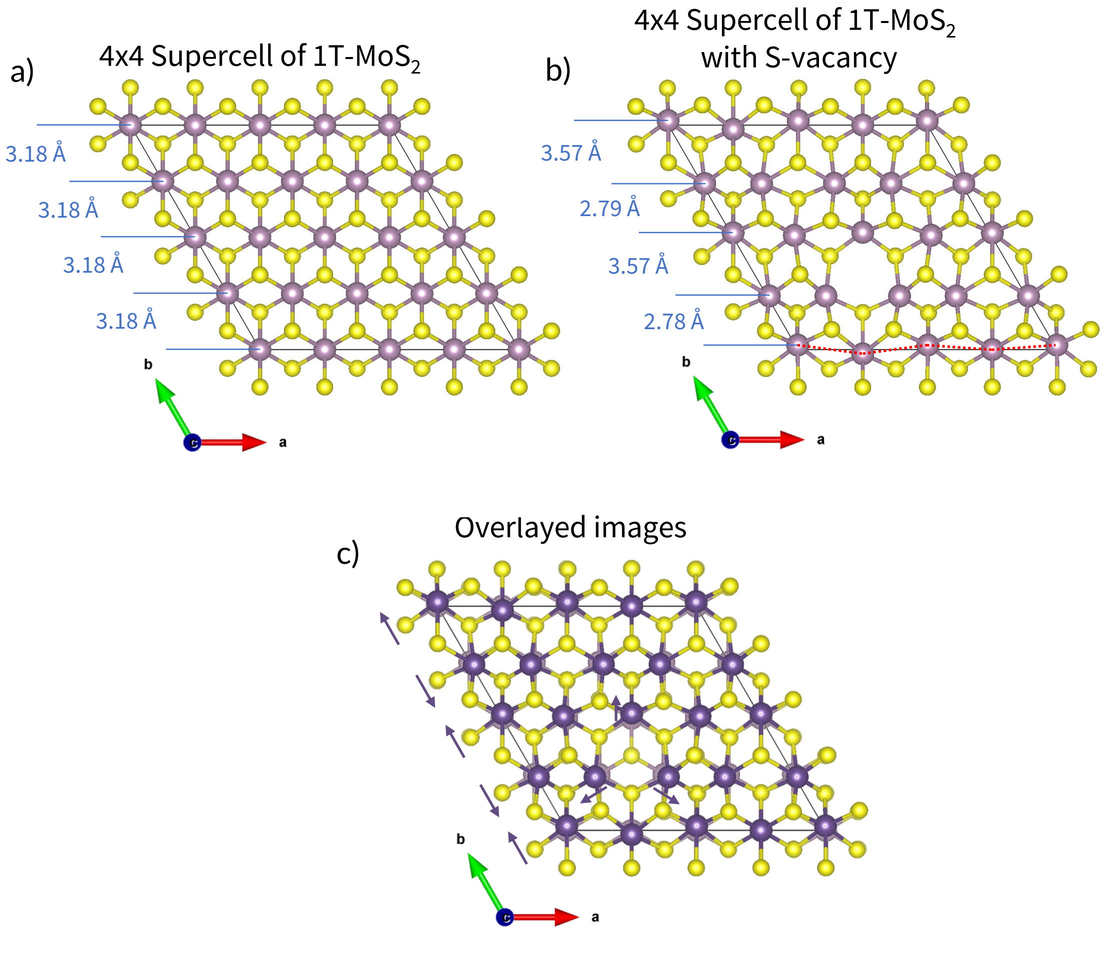

Figure S1: Top down view of 4x4 supercells of MoS_2 showing structural relaxations after introduction of a sulfur vacancy (sulfur atoms are yellow and molybdenum atoms are purple). (a) 4x4 supercell before a vacancy is introduced in the 1T-phase (b) 4x4 supercell after a vacancy is introduced showing dimerization along the b-axis. (c) Overlayed images of 1T-MoS_2 with (Mo-atoms in darker purple) and without a vacancy (Mo-atoms in lighter purple) with arrows showing the direction of some Mo atoms upon relaxation.

S1.2 Selection of Relaxation Radius

A relaxation radius (radius from center of vacancy in wich atoms were allowed to relax) was selected after observing that if the adsorbed intermediate structures were allowed to fully relax, unphysical distortions of the MoS_2 lattice would occur causing unrealistic deviations in energy along the pathway. Examples of these distortions are illustrated for two selected intermediates in Figure S2.

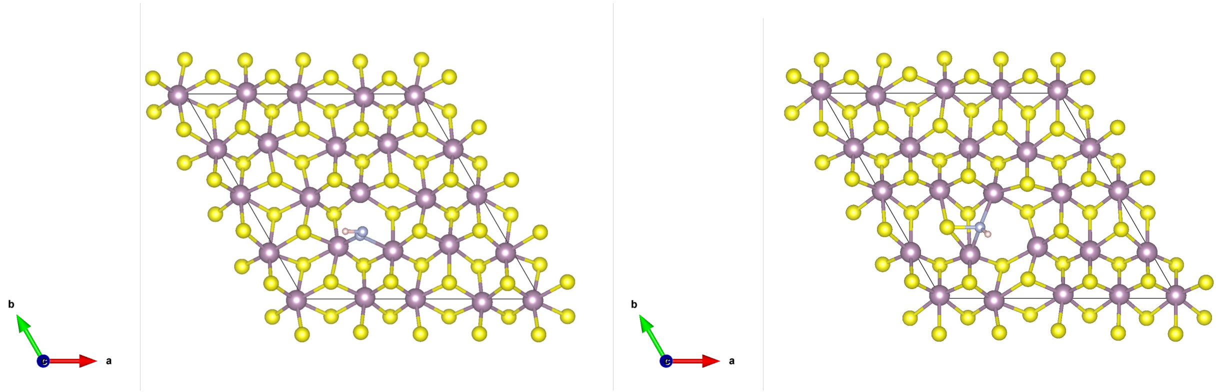

Figure S2: Top down view of 4x4 supercells of MoS_2 showing structural distortions of MoS_2 lattice when all atoms are allowed to relax for two selected intermediates.

To verify the effect of the size of the relaxation radius on the adsorption energy, we calculated the N_2 adsorption energy for different sized radii. For N_2 adsorption, we show that the adsorption energy fluctuates within 0.04 eV and the main effect of the relaxation radius is to prevent lattice distortions.

Table S1: N_2 adsorption energies for different relaxation radius sizes.

**Relaxation Radius (Å)**	$\mathbf{E_{ads}}$ **(eV)**
0	-0.29
2.0	-0.29
2.5	-0.29
3.0	-0.29
3.5	-0.30
4.0	-0.30
4.5	-0.29
5.0	-0.29
None	-0.33

S1.3 Adsorption site sampling

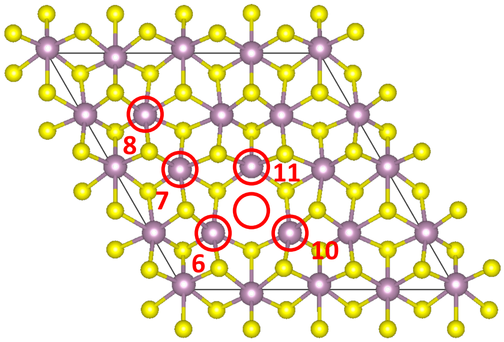

Figure S3: Top down view of 1T′-MoS_2-Sv structure. Red circles indicate N_2 binding sites that were sampled. E_{ads} values given in Table S2 with a note on final adsorption geometries.

Table S2: Table of initial N_2 adsorption geometries explored where $N_{2,z}$ indicates an out of plane orientation and $N_{2,x}$ indicates an in plane orientation.

Initial Orientation @ Site	E_{ads} (eV)	Final geometry
**$N_{2,z}$ @ Mo6**	-0.31	adsorbed
**$N_{2,z}$ @ Mo10**	-0.31	adsorbed
**$N_{2,z}$ @ Mo11**	-0.31	adsorbed
**$N_{2,x}$ @ Mo6+10**	-0.31	adsorbed to Mo6
**$N_{2,x}$ @ Mo6**	+0.49	adsorbed to Mo 10+11
**$N_{2,z}$ @ Center**	-0.23	In center
**$N_{2,z}$ @ Mo7**	-0.08	desorbed
**$N_{2,z}$ @ Mo8**	-0.09	desorbed

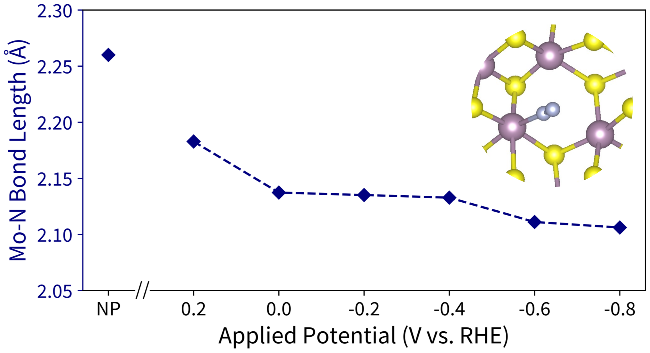

Figure S4: Mo–N bond length in the 1T′-MoS_2-Sv+N_2 structure under solvation with no potential in the canonical ensemble (NP) and applied potentials using GC-DFT

S2 Chemical equations for NRR pathways

S2.1 Standard pathways with NH_3 desorption

Alternating pathway

$$* + \mathrm{N_2} + 6\,(\mathrm{H^+} + \mathrm{e^-}) \rightleftharpoons {}^*\mathrm{N_2} + 6\,(\mathrm{H^+} + \mathrm{e^-}) \tag{1.1}$$
$${}^*\mathrm{N_2} + 6\,(\mathrm{H^+} + \mathrm{e^-}) \rightleftharpoons {}^*\mathrm{NNH} + 5\,(\mathrm{H^+} + \mathrm{e^-}) \tag{1.2}$$
$${}^*\mathrm{NNH} + 5\,(\mathrm{H^+} + \mathrm{e^-}) \rightleftharpoons {}^*\mathrm{NHNH} + 4\,(\mathrm{H^+} + \mathrm{e^-}) \tag{1.3A}$$
$${}^*\mathrm{NHNH} + 4\,(\mathrm{H^+} + \mathrm{e^-}) \rightleftharpoons {}^*\mathrm{NHNH_2} + 3\,(\mathrm{H^+} + \mathrm{e^-}) \tag{1.4A}$$
$${}^*\mathrm{NHNH_2} + 3\,(\mathrm{H^+} + \mathrm{e^-}) \rightleftharpoons {}^*\mathrm{NH_2NH_2} + 2\,(\mathrm{H^+} + \mathrm{e^-}) \tag{1.5A}$$
$${}^*\mathrm{NH_2NH_2} + 2\,(\mathrm{H^+} + \mathrm{e^-}) \rightleftharpoons {}^*\mathrm{NH_2} + \mathrm{NH_3} + 1\,(\mathrm{H^+} + \mathrm{e^-}) \tag{1.6A}$$
$${}^*\mathrm{NH_2} + \mathrm{NH_3} + 1\,(\mathrm{H^+} + \mathrm{e^-}) \rightleftharpoons {}^*\mathrm{NH_3} + \mathrm{NH_3} \tag{1.7}$$
$${}^*\mathrm{NH_3} + \mathrm{NH_3} \rightleftharpoons 2\,\mathrm{NH_3} \tag{1.8}$$

Distal pathway

$$* + \mathrm{N_2} + 6\,(\mathrm{H^+} + \mathrm{e^-}) \rightleftharpoons {}^*\mathrm{N_2} + 6\,(\mathrm{H^+} + \mathrm{e^-}) \tag{1.1}$$
$${}^*\mathrm{N_2} + 6\,(\mathrm{H^+} + \mathrm{e^-}) \rightleftharpoons {}^*\mathrm{NNH} + 5\,(\mathrm{H^+} + \mathrm{e^-}) \tag{1.2}$$
$${}^*\mathrm{NNH} + 5\,(\mathrm{H^+} + \mathrm{e^-}) \rightleftharpoons {}^*\mathrm{NNH_2} + 4\,(\mathrm{H^+} + \mathrm{e^-}) \tag{1.3D}$$
$${}^*\mathrm{NNH_2} + 4\,(\mathrm{H^+} + \mathrm{e^-}) \rightleftharpoons {}^*\mathrm{NNH_3} + 3\,(\mathrm{H^+} + \mathrm{e^-}) \tag{1.4D}$$
$${}^*\mathrm{NNH_3} + 3\,(\mathrm{H^+} + \mathrm{e^-}) \rightleftharpoons {}^*\mathrm{NH} + \mathrm{NH_3} + 2\,(\mathrm{H^+} + \mathrm{e^-}) \tag{1.5D}$$
$${}^*\mathrm{NH} + 2\,(\mathrm{H^+} + \mathrm{e^-}) \rightleftharpoons {}^*\mathrm{NH_2} + \mathrm{NH_3} + 1\,(\mathrm{H^+} + \mathrm{e^-}) \tag{1.6D}$$
$${}^*\mathrm{NH_2} + \mathrm{NH_3} + 1\,(\mathrm{H^+} + \mathrm{e^-}) \rightleftharpoons {}^*\mathrm{NH_3} + \mathrm{NH_3} \tag{1.7}$$
$${}^*\mathrm{NH_3} + \mathrm{NH_3} \rightleftharpoons 2\,\mathrm{NH_3} \tag{1.8}$$

S2.2 Pathways modified for NH_4^+ desorption in the final step

Alternating pathway

$$* + N_2 + 6\,(H^+ + e^-) + H^+ \rightleftharpoons {}^*N_2 + 6\,(H^+ + e^-) \tag{2.1}$$
$${}^*N_2 + 6\,(H^+ + e^-) + H^+ \rightleftharpoons {}^*NNH + 5\,(H^+ + e^-) \tag{2.2}$$
$${}^*NNH + 5\,(H^+ + e^-) + H^+ \rightleftharpoons {}^*NHNH + 4\,(H^+ + e^-) \tag{2.3A}$$
$${}^*NHNH + 4\,(H^+ + e^-) + H^+ \rightleftharpoons {}^*NHNH_2 + 3\,(H^+ + e^-) \tag{2.4A}$$
$${}^*NHNH_2 + 3\,(H^+ + e^-) + H^+ \rightleftharpoons {}^*NH_2NH_2 + 2\,(H^+ + e^-) \tag{2.5A}$$
$${}^*NH_2NH_2 + 2\,(H^+ + e^-) + H^+ \rightleftharpoons {}^*NH_2 + NH_3 + 1\,(H^+ + e^-) \tag{2.6A}$$
$${}^*NH_2 + NH_3 + 1\,(H^+ + e^-) + H^+ \rightleftharpoons {}^*NH_3 + NH_3 \tag{2.7}$$
$${}^*NH_3 + NH_3 + H^+ \rightleftharpoons NH_3 + NH_4{}^+ \tag{2.8}$$

Distal pathway

$$* + N_2 + 6\,(H^+ + e^-) + H^+ \rightleftharpoons {}^*N_2 + 6\,(H^+ + e^-) \tag{2.1}$$
$${}^*N_2 + 6\,(H^+ + e^-) + H^+ \rightleftharpoons {}^*NNH + 5\,(H^+ + e^-) \tag{2.2}$$
$${}^*NNH + 5\,(H^+ + e^-) + H^+ \rightleftharpoons {}^*NNH_2 + 4\,(H^+ + e^-) \tag{2.3D}$$
$${}^*NNH_2 + 4\,(H^+ + e^-) + H^+ \rightleftharpoons {}^*NNH_3 + 3\,(H^+ + e^-) \tag{2.4D}$$
$${}^*NNH_3 + 3\,(H^+ + e^-) + H^+ \rightleftharpoons {}^*NH + NH_3 + 2\,(H^+ + e^-) \tag{2.5D}$$
$${}^*NH + 2\,(H^+ + e^-) + H^+ \rightleftharpoons {}^*NH_2 + NH_3 + 1\,(H^+ + e^-) \tag{2.6D}$$
$${}^*NH_2 + NH_3 + 1\,(H^+ + e^-) + H^+ \rightleftharpoons {}^*NH_3 + NH_3 \tag{2.7}$$
$${}^*NH_3 + NH_3 + H^+ \rightleftharpoons NH_3 + NH_4{}^+ \tag{2.8}$$

S3 Calculated NRR pathways

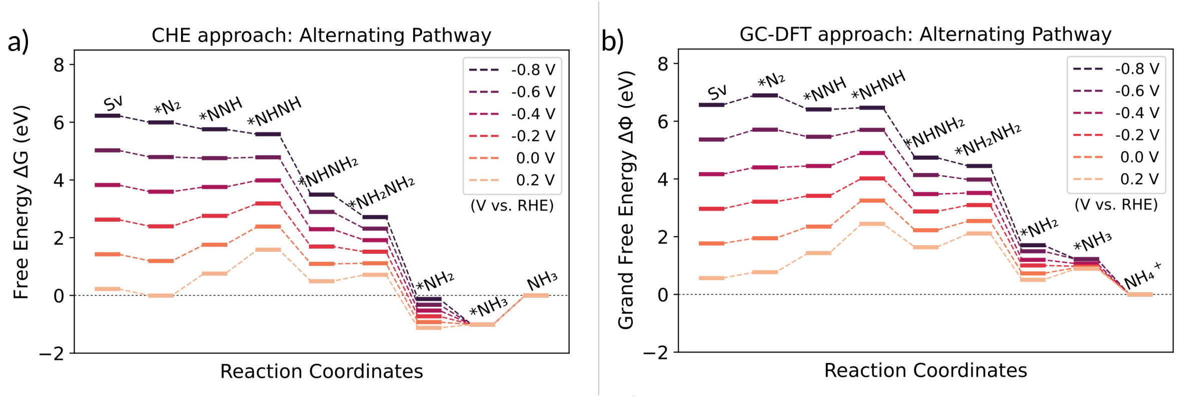

Figure S5: Alternating reaction pathway energetics in the CHE (a) and GC-DFT approach (b).

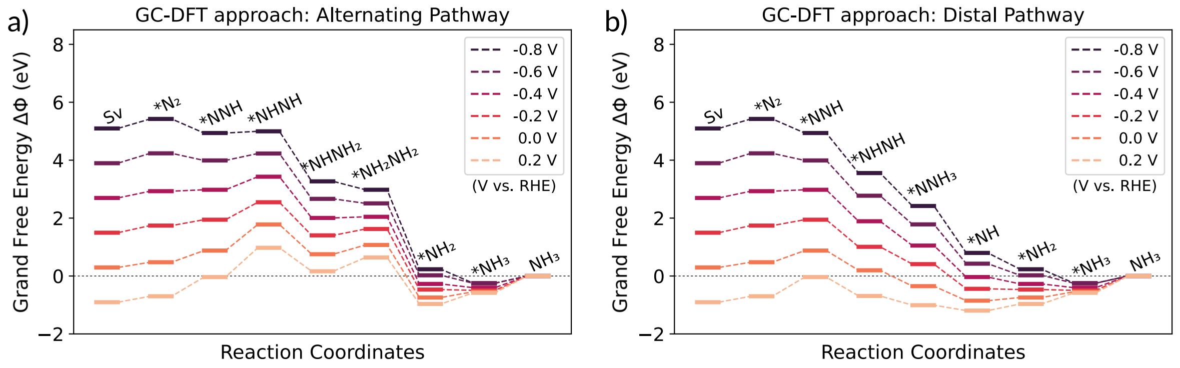

Figure S6: GC-DFT pathways referenced to NH_3 instead of $NH4^+$ (as in the main text) for the distal (a) and alternating (b) reaction pathways.

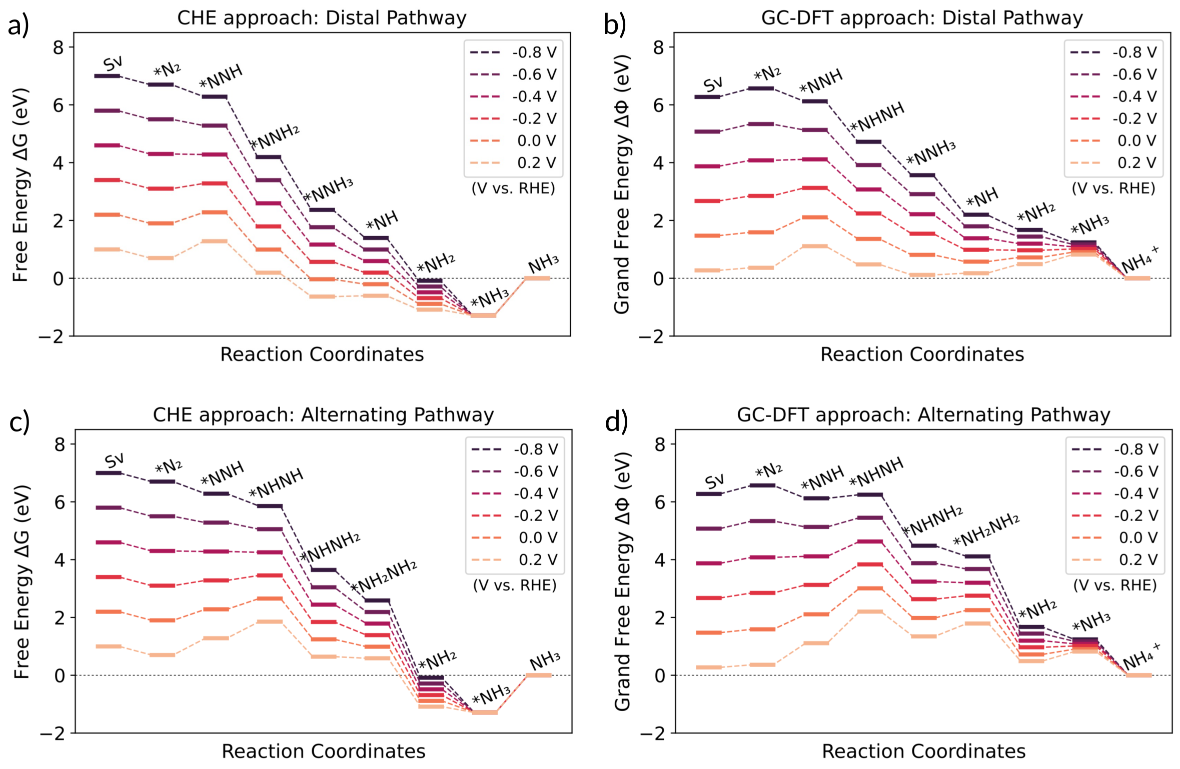

Figure S7: Energetics without vibrational corrections for the distal (a,b) and alternating (c,d) pathways in the CHE approach (a,c) and the GC-DFT approach (b,d).

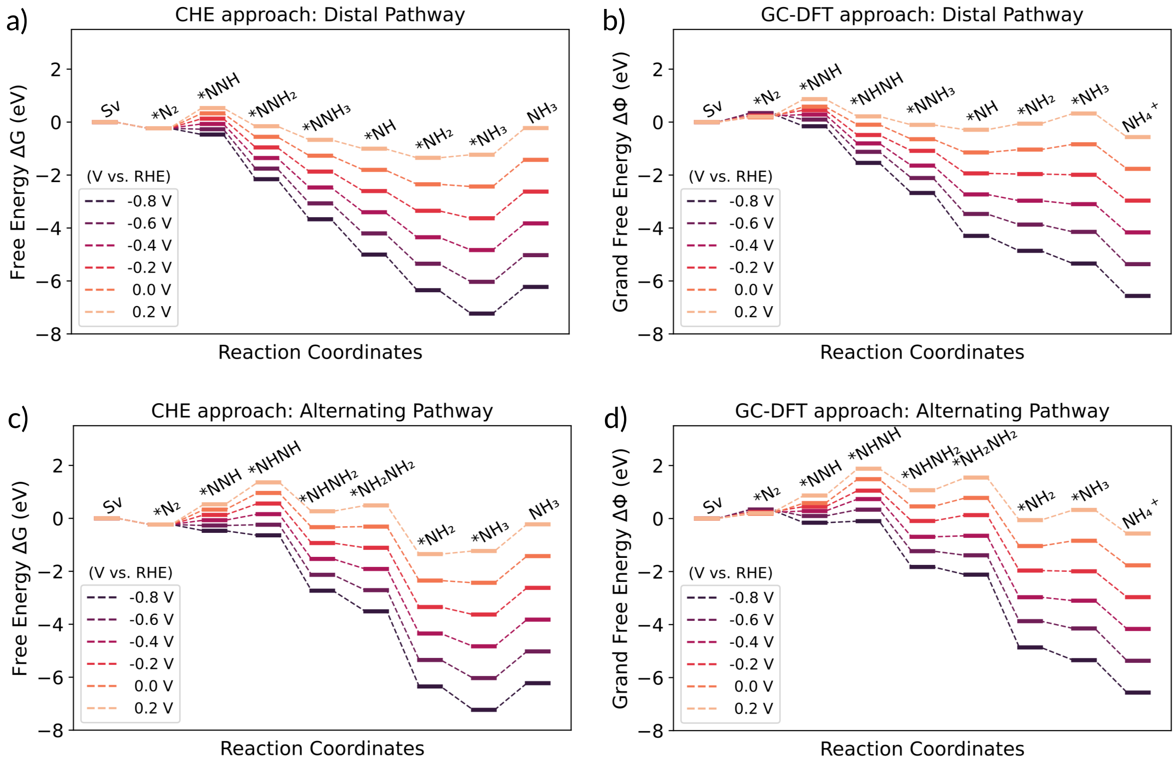

Figure S8: Pathways referenced to the initial rather than final state for the distal (a,b) and alternating (c,d) pathways in the CHE approach (a,c) and the GC-DFT approach (b,d). Note: This is exactly the same data shown in the main text, only the reference state has been changed.

S4 GC-NEB calculations for the $*N_2$ to *NNH step

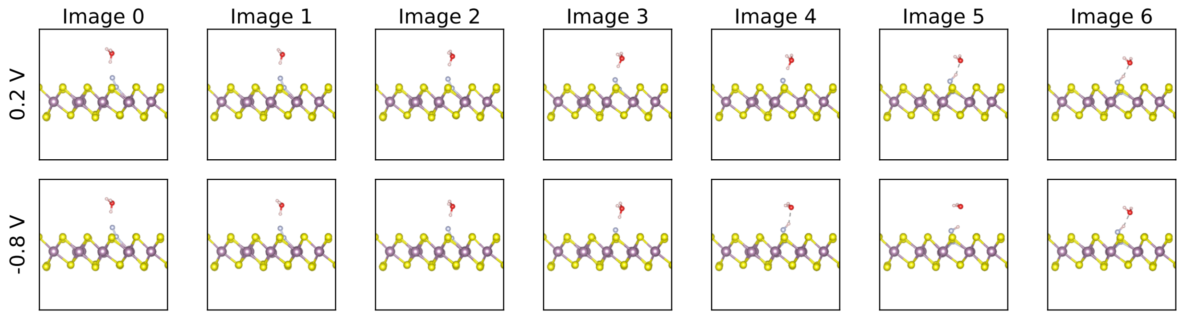

Figure S9: Pictures of converged structures of initial state, final state, and images obtained from GC-NEB calculations of the $*N_2$ to *NNH step under applied potential.

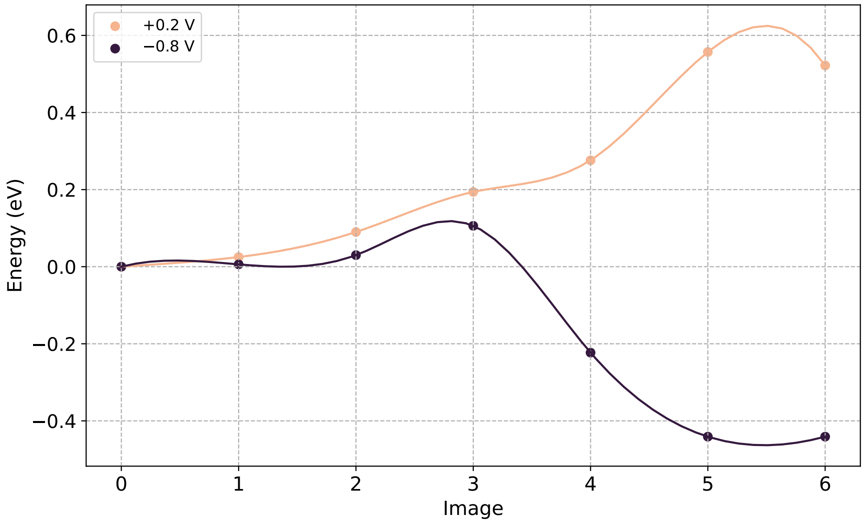

Figure S10: Computed GC-NEB energetics referenced to the initial state for the $*N_2$ to *NNH step.

S5 Calculated number of electrons in NRR reaction pathways

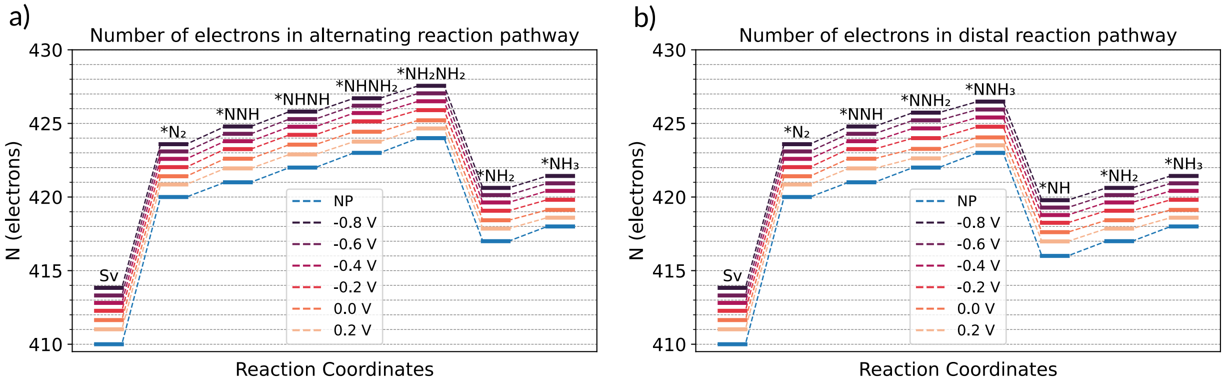

Figure S11: Calculated number of electrons along reaction path for the alternating reaction pathway (a) and distal reaction pathway (b) under no applied potential and under applied potential via GC-DFT showing all applied potentials charge the system more negatively than under the no potential condition.

S6 Structures and charge density difference plot for CO adsorption

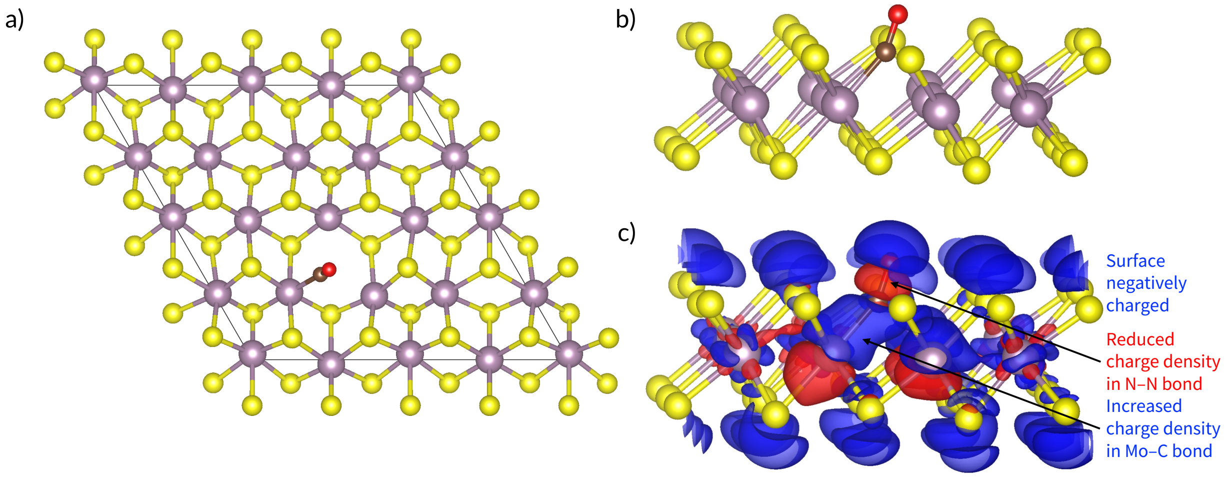

Figure S12: CO adsorption structures in top down view (a) and side on view (b,c) where (c) shows the charge density difference at the $\pm 0.0001\ e/\text{Å}^3$ isosurface level for a -0.2 V step towards negative potential (0.0 to -0.2 V shown, however, similar across all potential steps). Red indicates regions of charge loss, blue indicates region of charge gain.

S7 Density of States and Crystal Orbital Hamilton Population analysis

In order to gain a deeper understanding of the orbital interactions involved in bonding, we analyzed the orbital projected density of states (pDOS) of the 1T′-MoS_2-Sv structure, the N_2, CO, and H at Mo adsorption structures shown in main text Figure 6, as well as free N_2 and CO. We also performed chemical-bonding analysis to obtain the projected Crystal Orbital Hamilton Population (pCOHP)[1,2] for the N_2 bound structure. The pDOS calculations were performed in JDFTx[3] using a Gaussian broadening (E_σ) of 0.001 Ha and additional smoothing was applied using a Gaussian filter ($\sigma = 5$). For all pDOS figures, the Fermi level was shifted to 0 eV. The pCOHP curves were obtained using the Local Orbital Basis Suite Towards Electronic-Structure Reconstruction (LOBSTER) v 4.1.0 package.[4,5] LOBSTER enables projection of plane wave basis sets onto localized orbitals and thus can give the bonding and antibonding contributions to the pDOS from adjacent atoms. Since LOBSTER calculations cannot currently be run on JDFTx outputs, we ran single point (fixed geometry) calculations using the Vienna ab initio simulation package (VASP)[6–8] on the optimized structures and number of electrons from JDFTx to obtain inputs for LOBSTER. Calculation settings in VASP were chosen to closely match our JDFTx calculations considering the use of projected augemented wave (PAW) pseudopotentials.[9] We performed spin-polarized density functional theory calculations using the PAW method, cutoff energy of 600 eV, and Perdew–Burke–Ernzerhof (PBE) exchange–correlation functional. A $3 \times 3 \times 1$ k-point mesh was used with convergence threshold set to 1×10^{-5} eV. Solvation effects were treated by using the implicit solvation model implemented in VASPsol.[10,11]

The pDOS plots for the neat 1T′-MoS_2-Sv and 1T′-MoS_2-Sv+H@Mo are shown in Figure S13 while the pDOS plots for 1T′-MoS_2-Sv+N_2 and 1T′-MoS_2-Sv+CO and their corresponding free molecules are shown in Figure S14. We find that the molecular 2π* orbital of free N_2 broadens and hybridizes with the Mo d-states due to adsorption. The adsorbed N_2 2σ*, 2π, and 3σ orbitals also hybridize with Mo d-states, but still remain relatively sharp. Similar electronic structure observations for N_2 adsorption to single atom catalysts have been made.[12,13] In contrast, this interaction is not observed for 1T′-MoS_2-Sv+H@Mo, while no significant change is observed in the Mo 4d and molecular 2p state overlap in the pDOS of CO bound to 1T′-MoS_2-Sv, which remains strongly adsorbed across potentials. To understand bonding within the 1T′-MoS_2-Sv+N_2, we also performed COHP analysis of the Mo-*N_A and *N_A-N_B interactions in the 1T′-MoS_2-Sv+N_2 structure (Figure S15). The COHP analysis shows that the region in the DOS where the 2σ*, 2π, and 3σ orbitals N 2p states appear below the Fermi level corresponds to bonding interactions between Mo and N while the broad states above the Fermi level have primarily antibonding character. Additionally, the 3σ region flips to antibonding when the interaction between the nitrogen atoms are considered, indicating the electron density is likely being donated to the Mo in this bond. In the highlighted region of the DOS in S14b, we note the overlap between the broad Mo 4d and the N 3σ states correlates with the favorable to unfavorable adsorption of nitrogen with potential. That is, there is more overlap in these states when adsorption is most favorable (under no applied potential), and less overlap when adsorption is least favorable (-0.8 V applied potential). Finally, since GGA level DFT tends to underestimate energy gaps, we validated these findings by running a single point hybrid calculation on each of the PBE structures using PBE0. Despite the increase in spread of the density of states, the overlap trends for the Mo 4d and

the N 3σ remained the same as shown in Figure S15. We conjecture that the large spread in energy of Mo 4d states is key to their ability to interact with bonding and antibonding states of backbonding molecules well above and below the Fermi energy.

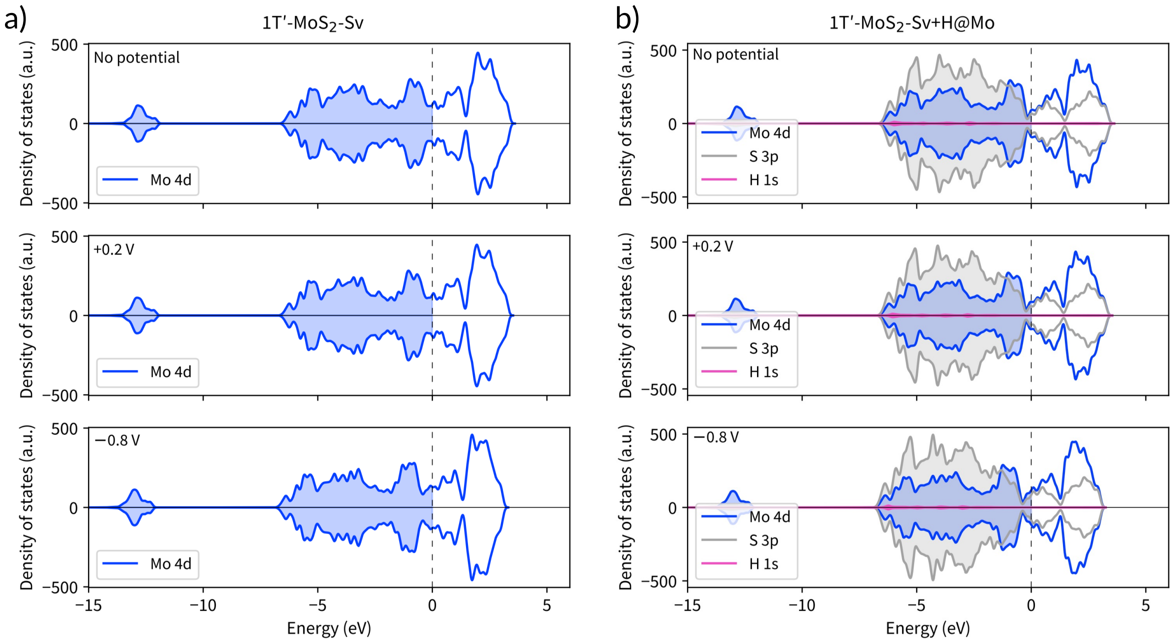

Figure S13: pDOS of (a) 1T′-MoS_2-Sv and (b) 1T′-MoS_2-Sv+H@Mo under no potential, +0.2 V, and −0.8 V conditions.

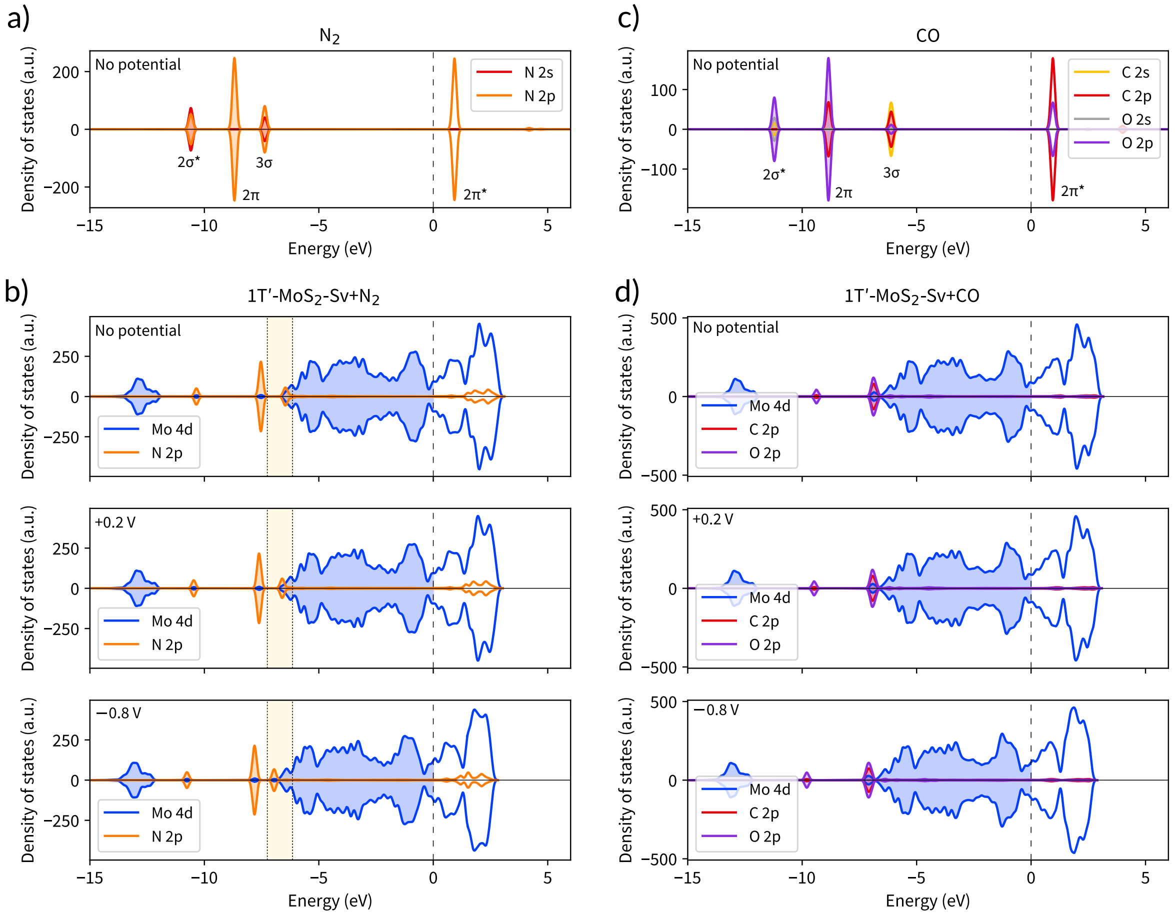

Figure S14: pDOS of (a) free N_2, (b) 1T′-MoS_2-Sv+N_2, (c) free CO, and (d) 1T′-MoS_2-Sv+CO under no potential, +0.2 V, and −0.8 V conditions for the bound structures.

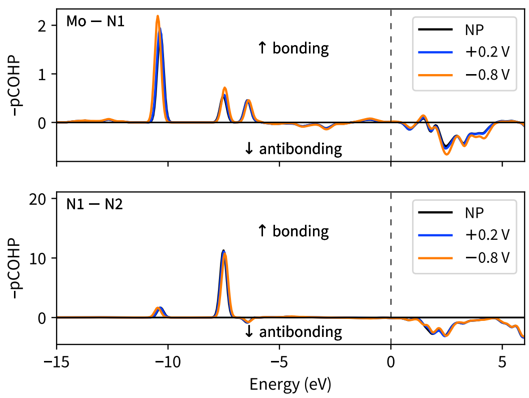

Figure S15: -pCOHP curves for Mo-N_A and N_A-N_B atom interactions showing bonding (positive) and antibonding (negative) states. N_A represents the nitrogen bound to the Mo site and N_B the outer nitrogen as defined in the main text.

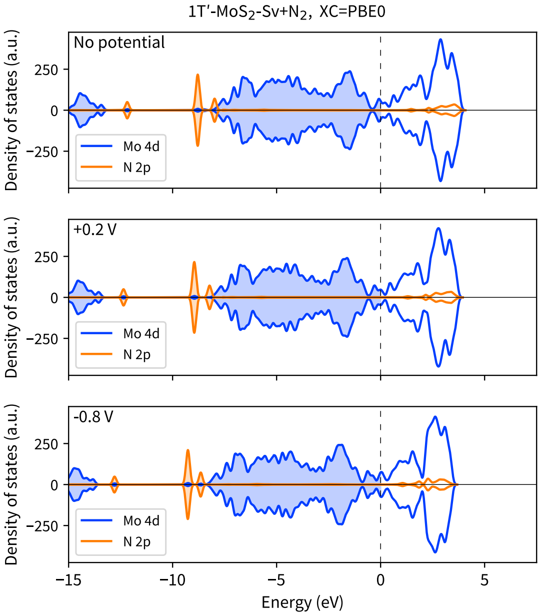

Figure S16: pDOS of 1T′-MoS_2-Sv+N_2 at the PBE0 level under no potential, +0.2 V and −0.8 V conditions.

References

(1) Deringer, V. L.; Tchougréeff, A. L.; Dronskowski, R. Crystal Orbital Hamilton Population (COHP) Analysis As Projected from Plane-Wave Basis Sets. *The Journal of Physical Chemistry A* **2011**, *115*, 5461–5466.

(2) Dronskowski, R.; Bloechl, P. E. Crystal Orbital Hamilton Populations (COHP): Energy-Resolved Visualization of Chemical Bonding in Solids Based on Density-Functional Calculations. *The Journal of Physical Chemistry* **1993**, *97*, 8617–8624.

(3) Sundararaman, R.; Letchworth-Weaver, K.; Schwarz, K. A.; Gunceler, D.; Ozhabes, Y.; Arias, T. A. JDFTx: Software for Joint Density-Functional Theory. *SoftwareX* **2017**, *6*, 278–284.

(4) Maintz, S.; Deringer, V. L.; Tchougréeff, A. L.; Dronskowski, R. Analytic Projection from Plane-Wave and PAW Wavefunctions and Application to Chemical-Bonding Analysis in Solids. *Journal of Computational Chemistry* **2013**, *34*, 2557–2567.

(5) Nelson, R.; Ertural, C.; George, J.; Deringer, V. L.; Hautier, G.; Dronskowski, R. LOBSTER: Local Orbital Projections, Atomic Charges, and Chemical-Bonding Analysis from Projector-Augmented-Wave-Based Density-Functional Theory. *Journal of Computational Chemistry* **2020**, *41*, 1931–1940.

(6) Kresse, G.; Hafner, J. Ab Initio Molecular Dynamics for Liquid Metals. *Physical Review B* **1993**, *47*, 558–561.

(7) Kresse, G.; Furthmüller, J. Efficiency of Ab-Initio Total Energy Calculations for Metals and Semiconductors Using a Plane-Wave Basis Set. *Computational Materials Science* **1996**, *6*, 15–50.

(8) Kresse, G.; Furthmüller, J. Efficient Iterative Schemes for Ab Initio Total-Energy Calculations Using a Plane-Wave Basis Set. *Physical Review B* **1996**, *54*, 11169–11186.

(9) Kresse, G.; Joubert, D. From Ultrasoft Pseudopotentials to the Projector Augmented-Wave Method. *Physical Review B* **1999**, *59*, 1758–1775.

(10) Mathew, K.; Sundararaman, R.; Letchworth-Weaver, K.; Arias, T. A.; Hennig, R. G. Implicit Solvation Model for Density-Functional Study of Nanocrystal Surfaces and Reaction Pathways. *The Journal of Chemical Physics* **2014**, *140*, 084106.

(11) Mathew, K.; Kolluru, V. S. C.; Mula, S.; Steinmann, S. N.; Hennig, R. G. Implicit Self-Consistent Electrolyte Model in Plane-Wave Density-Functional Theory. *The Journal of Chemical Physics* **2019**, *151*, 234101.

(12) Lv, X.; Wei, W.; Huang, B.; Dai, Y.; Frauenheim, T. High-Throughput Screening of Synergistic Transition Metal Dual-Atom Catalysts for Efficient Nitrogen Fixation. *Nano Letters* **2021**, *21*, 1871–1878.

(13) Bo, T.; Cao, S.; Mu, N.; Xu, R.; Liu, Y.; Zhou, W. High-Throughput Screening of Transition Metal Single-Atom Catalysts for Nitrogen Reduction Reaction. *Applied Surface Science* **2023**, *612*, 155916.
